# A root functional–structural model allows assessment of the effects of water deficit on water and solute transport parameters

**DOI:** 10.1093/jxb/erac471

**Published:** 2022-12-14

**Authors:** Fabrice Bauget, Virginia Protto, Christophe Pradal, Yann Boursiac, Christophe Maurel

**Affiliations:** Institute for Plant Sciences of Montpellier (IPSiM), Univ Montpellier, CNRS, INRAE, Institut Agro, Montpellier, France; Institute for Plant Sciences of Montpellier (IPSiM), Univ Montpellier, CNRS, INRAE, Institut Agro, Montpellier, France; CIRAD, UMR AGAP Institute, Montpellier, France; Inria & LIRMM, Univ Montpellier, CNRS, Montpellier, France; Institute for Plant Sciences of Montpellier (IPSiM), Univ Montpellier, CNRS, INRAE, Institut Agro, Montpellier, France; Institute for Plant Sciences of Montpellier (IPSiM), Univ Montpellier, CNRS, INRAE, Institut Agro, Montpellier, France; Ikerbasque, Spain

**Keywords:** Conductance, model, root hydraulics, root system architecture, solute transport, water deficit, water transport, xylem

## Abstract

Root water uptake is driven by a combination of hydrostatic and osmotic forces. Water transport was characterized in primary roots of maize seedlings grown hydroponically under standard and water deficit (WD) conditions, as induced by addition of 150 g l^–1^ polyethylene glycol 8000 (water potential= –0.336 MPa). Flow measurements were performed using the pressure chamber technique in intact roots or on progressively cut root system architectures. To account for the concomitant transport of water and solutes in roots under WD, we developed within realistic root system architectures a hydraulic tree model integrating both solute pumping and leak. This model explains the high spontaneous sap exudation of roots grown in standard conditions, the non-linearity of pressure–flow relationships, and negative fluxes observed under WD conditions at low external hydrostatic pressure. The model also reveals the heterogeneity of driving forces and elementary radial flows throughout the root system architecture, and how this heterogeneity depends on both plant treatment and water transport mode. The full set of flow measurement data obtained from individual roots grown under standard or WD conditions was used in an inverse modeling approach to determine their respective radial and axial hydraulic conductivities. This approach allows resolution of the dramatic effects of WD on these two components.

## Teaser

A model integrating water and solute transport within realistic root system architectures allows assessment of the effects of water deficit on elementary root transport parameters.

## Introduction

The uptake of soil water by roots is crucial for the growth and survival of most terrestrial plants. Root water uptake allows the plant to maintain its water status, under favorable or adverse environmental conditions such as drought, by continuously balancing transpirational water losses and water required for expansion growth (Steudle, 2000a). Root water uptake sequentially involves a radial transport of water from the soil to the root stele and its axial transport along the root vasculature. Radial transport is mediated through cell wall (apoplastic) and cell-to-cell (symplastic or transcellular) pathways running across peripheral cell layers (epidermis, cortex, and endodermis) down to the xylem. Transcellular water transport is mediated in large part by aquaporin (AQP) water channels (Maurel *et al.*, 2015). Axial transport consists of conveying sap flow along xylem vessels, throughout the whole root system architecture (RSA), up to the aerial parts (Gambetta *et al.*, 2017). The conductance of the radial and axial pathways can be altered in response to multiple environmental cues, providing a continuous adjustment of root hydraulics to water availability and demand (Aroca *et al.*, 2011; Boursiac *et al.*, 2022b). In particular, water deprivation can lead to rapid changes in AQP activity (Hachez *et al.*, 2012). These effects which depend on drought intensity can result, depending on genetic background, in a monotonous inhibition of root hydraulic conductivity (*L*p_r_) or in bell-shaped dose–response curves (Rosales *et al.*, 2019). In the longer term, water deficit (WD) can also alter xylem development, with contrasting effects depending on the species (Ramachandran *et al.*, 2020). Finally, WD enhances suberization of both the exo- and endodermis, possibly altering solute transport (Doblas *et al.*, 2017).

In recent years, efforts have been made to assess the radial and axial water transport properties of plant roots, using a combination of experimental and mathematical modeling approaches on excised segments or whole root systems (Zarebanadkouki *et al.*, 2016; Meunier *et al.*, 2018; Heymans *et al.*, 2021; Boursiac *et al.*, 2022a). Most recent works rely on hydraulic models embedded within RSA, based on the hydraulic tree model of Doussan *et al.* (Doussan *et al.*, 1998a, b; Zarebanadkouki *et al.*, 2016; Meunier *et al.*, 2017; Boursiac *et al.*, 2022a), or on approaches that combine a hydraulic cross-sectional model at the cellular level with anatomical observations along the root (Couvreur *et al.*, 2018; Ding *et al.*, 2020; Heymans *et al.*, 2021).

More integrative root models that couple water and solute transport have also existed for decades. The first models were based on a representation of the root as an osmometer. The whole root system was reduced to one, two, or even three membranes separating homogeneous compartments (Fiscus, 1975, 1977, 1986; Miller, 1985b; Steudle, 1994; Murphy, 2000). Some more recent models provide a longitudinal, bi-dimensional representation of unbranched root structures considering the role and transport selectivity of Casparian strips and suberin lamellae (Foster and Miklavcic, 2016, 2017). Complementary to these, Couvreur *et al.* (2018) proposed a 2D cross-sectional model computing water transport at subcellular levels (walls, membranes, and plasmodesmata) and considering apoplastic solute diffusion and symplastic homeostasis.

Hydraulic models at the RSA level are designed to account for the non-uniformity of hydraulic parameters such as axial conductance. Therefore, they can efficiently simulate the heterogeneity, over the whole root system, of radial and axial hydraulic flows and of water potential components (e.g. hydrostatic pressure in the xylem). However, none of these models has been able to simulate whole root water transport under WD conditions, when osmotic driving forces become predominant. Current root osmometer models can do so but, conversely, cannot represent the heterogeneous aspects of an RSA since the root is modeled as a restricted number of compartments in which solute concentration is homogeneous and pressure is uniform. Interestingly, these models have been able to mimic to a certain extent some experimental and puzzling observations such as the non-linear relationship between sap flow and pressure (Steudle, 1994). Integrating water and solute transport models within RSA could therefore provide a means to account for all the pre-cited aspects and, ultimately, investigate the multiple effects of WD on root water and solute transport parameters.

Boursiac *et al.* (2022a) recently proposed an inverse modeling approach, based on pressure chamber measurements in excised roots, to simultaneously assess axial conductance and radial conductivity of complex branched root structures. This approach, called cut-and-flow, consists of measuring the exuding sap flow rate, at a constant working pressure, in a root system that is successively cut from its distal part. A hydraulic tree model named HydroRoot (available at Zenodo, doi: 10.5281/zenodo.6584200) was inverted to fit the cut-and-flow data, thereby allowing a determination of the axial conductance profile and radial conductivity on the same root system. Because it exclusively considers hydrostatic driving forces, this model cannot operate in roots under WD or when osmotic forces are predominant.

To fill this gap and describe the concomitant transport of water and solutes in roots under WD, we coupled within realistic RSAs of maize seedlings a hydraulic model to a solute transport model of the Fiscus type (Fiscus, 1977). Cut-and-flow data and pressure–flow relationships [*J*_v_(*P*)] were used in an inverse modeling approach to determine the radial and axial hydraulic conductivities of primary roots grown under standard or WD conditions. This approach allows resolution of the dramatic effects of WD on both radial and axial conductivities.

## Materials and methods

### Plant material, growth, and experimental conditions

Seeds of a maize B73-UH007 hybrid (B73H) (Millet *et al.*, 2016) were surface-sterilized in 50 ml of 1.4% bleach with a drop (~50 µl) of Tween-20 for 15 min under gentle agitation. The seeds were then treated with 35% H_2_O_2_ for 2 min, rinsed with 70% ethanol, and washed six times with sterilized water. The seeds were overlaid with wet clay beads in a plastic box, which was itself covered by a transparent plastic film. Seeds were germinated in the dark and further grown in a growth chamber at 65% relative humidity, with 22 °C/20 °C and 15 h/9 h light/dark cycles (150 µmol m^–2^ s^–1^).

At 5 days after sowing (DAS), seedlings were transferred to a hydroponic container filled with 24 liters of a medium containing 1.25 mM KNO_3_, 0.75 mM MgSO_4_, 1.5 mM Ca(NO_3_)_2_, 0.5 mM KH_2_PO_4_, 0.1 mM MgCl_2_, 0.05 mM Fe-EDTA, 0.05 mM H_3_BO_3_, 0.012 mM MnSO_4_, 0.7 mM CuSO_4_, 0.001 mM ZnSO_4_, 24 × 10^–5^ mM MoO_4_Na_2_, 1 × 10^–5^ mM CoCl_2_, 0.1 mM Na_2_SiO_3_, and 1 mM MES. Plants were grown in this solution for 2 d. At 7 DAS, seedlings were transferred for an additional 4 d to a fresh medium containing 150 g l^–1^ of high molecular weight polyethylene glycol (PEG 8000) to reduce the water potential of the nutrient solution. The control plants were transferred for 4 d in a fresh hydroponic solution. The water potential of the control hydroponic solution (–0.034 MPa) was measured with a WESCOR 5520 vapor pressure osmometer, with a resolution of 1 mmol kg^–1^ (2.5 × 10^–3^ MPa at 293 °K). Addition of 150 g l^–1^ PEG to the control solution leads to a final water potential of –0.336 MPa, according to the empiric law used to determine the water potential of PEG solution (see below ‘Viscosity and water potential of a PEG solution’).

Overall, two bathing solutions were used for plant growth and pressure chamber experiments: the control (CTR) hydroponic solution and the hydroponic solution containing 150 g l^–1^ PEG (PEG). This resulted in three plant sets: (i) eight CTR plants, grown in a CTR hydroponic solution, with measurements done in the same solution; (ii) eight PEG plants grown in a PEG hydroponic solution, with measurements done in the same solution; and (iii) five PEG-CTR plants grown in a PEG hydroponic solution, and transferred into a CTR solution for 1 h prior to water transport measurements in the same CTR solution.

### Measurements of pressure-dependent xylem sap flow [*J*_v_(*P*)]

Root water flow was measured on de-topped primary roots using a set of pressure chambers with automated recording as described in Boursiac *et al.* (2022a). The primary root was carefully excised below the grain, placed into an adapter sealed with dental paste (Coltene Whaledent, France), and threaded across the silicone seal of the pressure chamber lid. The root was then placed into the pressure chamber in a container filled with either a CTR or PEG hydroponic solution. The adapter was connected to a flowmeter (Bronkhorst, France) in order to record the flow of sap (*J*_v_) from the root system at successive pressures (*P*) applied on the bathing solution using nitrogen gas. Typical successive relative pressure steps were, in MPa: 0.00, 0.05, 0.15, 0.10, 0.25, 0.20, 0.35, 0.30, 0.45, 0.50, 0.25, and 0.00 (0.00 represents the atmospheric pressure). Some experimental datasets comprise fewer pressure steps, down to four including exudation at atmospheric pressure. Only four plants of 21 had fewer than six steps.

To estimate the deviation of a measured or adjusted *J*_v_(*P*) relationship from a linear response to pressure, we normalized between 0 and 1 the fluxes and pressures according to their minima and maxima. This allows comparison of roots that have very disparate fluxes in absolute values. We then quantified the gap to linearity by the maximal residual between the *J*_v_(*P*) relationship and the bisector.

### Cut-and-flow experiment

Cut-and-flow measurements were performed just after *J*_v_(*P*) measurements. The operating pressure was set constant over the whole experiment: 0.3 MPa with PEG solution and 0.2 MPa with CTR solution (except for two plants done at 0.3 MPa instead of 0.2 MPa). In brief, and along the lines of our previous work (Boursiac *et al.*, 2022a), *J*_v_ was first recorded in the intact root system at the operating pressure. The root was taken out of the chamber and placed in a Petri dish filled with bathing solution. The lateral roots were stretched and aligned along the primary root and the whole root system was cut with a razor blade at a given distance to the tip, typically a few centimeters. Leaving the cut root segments in the Petri dish, the remaining part of the root system was placed back into the chamber at the operating pressure and the *J*_v_ was recorded. This procedure was repeated successively.

To be as fast as possible during the cutting process (average cutting time was 140 ± 8 s), the cut length between successive excisions was approximate. Overall, the first cuts were 8.3 ± 0.6 cm on average whereas the mean length of subsequent cuts was 4.3 ± 0.2 cm and the average length of the remaining root system was 5.2 ± 0.6 cm.

### Digitizing of root architectures

The root segments released after each cut and the basal root system remaining after the last cut were scanned at 600 dpi. The root parts were then digitized using SmartRoot (Lobet *et al.*, 2011), an ImageJ plugin; the segment lengths and lateral root positions on their parent root were exported as a text file in csv format. The complete root system was then reconstructed by recognizing, at each cut, the segments belonging to the primary root and those belonging to the laterals, and by pasting them to the remaining system. For example, considering an experiment with *n* successive cuts, the segments from the last cut were pasted to the remaining basal root system. Then the segments from the previous cut (*n*–1) were pasted to this new basal root system, and so on. Note that we did not scan the intact root system to avoid any damage before measurements.

The diameters of the primary root and its laterals were entered into the model for building the representation of the RSA (see ‘Water and solute transport model’). They were determined from three independent panels of CTR and PEG plants. Each experiment had 6–10 plants of each type, yielding a final count of 23 CTR and 28 PEG plants. The roots were scanned, and the diameters were estimated using ImageJ. The average primary root diameters were 1.05 mm (*n*=104) and 1.03 mm (*n*=133) for CTR and PEG plants, respectively, with SEs in the range of 2 × 10^–3^ mm. The average lateral root diameters were 0.36 mm (*n*=1345) and 0.39 mm (*n*=1495) for CTR and PEG plants, respectively, with SEs <2 × 10^–4^ mm. The 11-day-old roots had only first-order laterals.

### Water and solute transport model

The present model is a modification of the HydroRoot RSA hydraulic model (Boursiac *et al.*, 2022a). HydroRoot was developed as a component of the OpenAlea platform (Pradal *et al.*, 2008). It uses a multiscale tree graph (MTG) (Godin and Caraglio, 1998) to represent a root hydraulic architecture, which consists of the topology of a root system (branching positions, root lengths, root radii, etc.) and its hydraulic structure (local radial and axial conductivities). With respect to Boursiac *et al.* (2022a), the main modification of HydroRoot was the addition of solute transport equations to the hydraulic model. This change led to a major difference in the resolution of the equation system on the whole RSA. Thus, the hydraulic architecture can no longer be modeled by an analogous electrical network (Boursiac *et al.*, 2022a) and the coupled solute and water transport equations had to be solved in a matrix form.

**Fig. 1.**
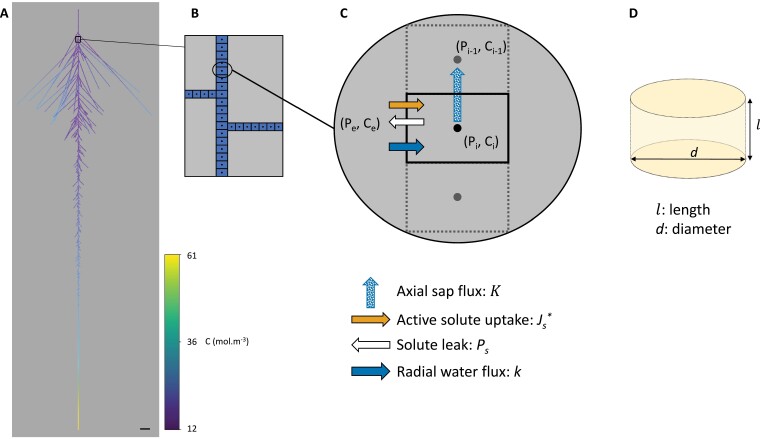
Modeling water and solute transport within realistic RSAs of maize primary roots. (A) RSA of a CTR root with a heat map representation of the solute concentration. Scale bar=10 mm. (B) The architecture was discretized in representative elementary volumes (REVs). (C) Sketch of the different fluxes in and out of a REV, for *P*_e_>*P*_i_>*P*_i–1_ and *C*_i_>*C*_e_, and in the absence of PEG. The sap flow along the xylem vessels is characterized by the axial conductance *K* profile. The water flow across the peripheral root tissues and into the xylem is characterized by the radial hydraulic conductivity *k*. The solutes are taken up into the xylem vessels at a constant rate *J*_s_*. *P*_s_ is the tissue permeability of the solutes. (D) REV geometry characterized by its length *l* and its diameter *d* that depends on the root order (axial or first-order lateral root).

The model was developed at millimetric scale, the order of magnitude of the primary root diameter. The RSA was discretized in cylindrical elementary volumes (Fig. 1), considered as representative elementary volumes (REVs). The REV diameter, *d*, is equal to the root diameter, and depends on the root order (primary or first laterals). The REV length, *l*, is of the order of the diameter, here 1 mm, which was small enough to obtain numerical convergence. The local transport equations described below were considered in each REV. Each REV can be seen as two concentric media: the peripheral tissues (from epidermis to pericycle) through which radial transport happens, and a central medium (stele with xylem vessels) where the sap flows axially. In the following, the parameter units are displayed in parentheses and correspond to the international system of units.

#### Water transport

The hydraulic model is the same as in HydroRoot, with the addition of a radial water flow rate due to osmotic potential difference. Thus, the radial water flux can be modeled as follows:


$$\begin{array}{l} j=k\left(\Delta \Psi_{H}+\Delta \Psi_{peg}+\sigma \Delta \Psi_{S}\right)S \end{array}$$ (1)

where *j* is the local radial water flow rate, *k* (m s^–1^ MPa^–1^) the radial hydraulic conductivity. ΔΨ_H_, ΔΨ_peg_, and ΔΨ_S_ (MPa) correspond to the hydrostatic water potential difference between the bathing solution and the xylem sap, to the osmotic water potential difference due to the PEG, and to the osmotic water potential difference due to the solutes, respectively. σ is the effective reflection coefficient. *S* (S=π*dl*) is the external surface area of the REV (m^2^). Expressing the water potentials, Equation 1 can be written as follows:


$$\begin{array}{l} j=k\left(P_{e}-P-\pi_{peg}^{ext}+\pi_{peg}-\sigma RT\left(C_{e}-C\right)\right)S \end{array}$$ (2)

where *P*_*e*_ and *P* are the hydrostatic pressure of the bathing solution and within the xylem vessels, respectively, $\pi_{peg}^{ext}$ and $\pi_{peg}$ are the PEG contribution to the osmotic pressure of the bathing solution and inside the xylem vessels, respectively, *C*_*e*_ and *C* are the solute concentration in the bathing solution and in the xylem vessels (mol m^–3^), respectively, *R* the gas constant, and *T* the temperature (set to 298 °K here). Both $\pi_{peg}^{ext}$ and $\pi_{peg}$ are calculated according to the PEG concentration (Supplementary Fig. S1A). Since PEG 8000 is a non-permeant solute, its contribution to the osmotic pressure inside the xylem ($\pi_{peg}$) is only considered in cut-and-flow experiments within PEG bathing solutions.

The axial sap flow rate was modeled with a Hagen–Poiseuille’s law type:


$$\begin{array}{l} J=K\left(µ\right)\frac{\Delta P}{l} \end{array}$$ (3)

where *J* is the axial sap flow rate, *K*(μ) the axial conductance that depends on sap viscosity (m^4^ s^–1^ MPa^–1^; see below), Δ*P* the local ­pressure ­difference between two REVs, and *l* the length of the REV. The axial conductance is inversely proportional to the fluid viscosity μ, as illustrated for example by the conductance in a cylindrical capillary of radius $r\;\;:K(µ)=\pi r^{4}/(8µ)$. The sap is commonly considered as having water viscosity. Thus, axial conductances were displayed as corresponding to a sap viscosity of 1 mPa s (Equation 3) and not independently of the viscosity, as follows: J=(K/µ) (ΔP/l). The former expression is commonly used in the literature (Landsberg and Fowkes, 1978; Doussan *et al.*, 1998a, b; Zarebanadkouki *et al.*, 2016; Meunier *et al.*, 2018; Heymans *et al.*, 2021) and allows better comparisons. Note, however, that when PEG penetrates the root vasculature in cut-and-flow experiments, the viscosity significantly increases with the PEG concentration (see next section) and has to be taken into account.

To reduce the number of parameters to be adjusted (see ‘Parameter determination’), σ was set to 0.85, a value reported for nutrients on maize root systems (Miller, 1985a). Sensitivity tests were done to evaluate the impact of σ on the other parameters (see the Results and Discussion).

#### Solute transport

The modeling of solute transport is based on two experimental observations: exudation at atmospheric pressure and negative sap flow observed in some PEG roots. Under atmospheric pressure monitoring, sap flow is driven by the osmotic pressure gradient due to the solute concentration difference between the outer solution and xylem sap (see Equation 2). Pure hydraulic models, without any solute transport to the xylem vessels, cannot explain such behavior in steady state. To account for a constant positive exudation rate, the solutes exiting the root by exudation must indeed be balanced by an uptake of solutes. Thus, in addition to the water radial flux, we hypothesized a constant active uptake rate of solutes, denoted *J*_s_* after Fiscus (Fiscus, 1975). Yet, the steady and negative sap flow rate observed in some PEG roots raises specific questions. If sap continuously flows from the root base to the bathing solution, whereas solutes driven by *J*_s_* keep entering the root at a constant rate, the solute concentration in xylem vessels should increase indefinitely, in contradiction to the mass balance principle that should be verified in any REV of the root. Therefore, an additional solute flux, or leakage, from the xylem vessels to the root bathing solution was modeled as a passive diffusion characterized by the tissue permeability *P*_s_. The radial solute flux was therefore expressed as follows:


$$\begin{array}{l} j_{s}=\left[\;J_{s}^{*}-P_{s}\left(C-C_{e}\right)\right]S \end{array}$$ (4)

where *j*_s_ (mol s^–1^) is the radial solute flux, *J*_s_* (mol m^–2^ s^–1^) is the solute active uptake rate, and *P*_s_ (m s^–1^) is the radial permeability of the root peripheral tissues. As in Equation 2, *C* and *C*_e_ correspond to the solute concentration in the xylem vessels and in the bathing solution, respectively. *S* is the external surface area of the REV.

Since solutes are transported along xylem vessels by advection, axial solute flux can be expressed as *J*_s_=*JC* (mol s^–1^). When PEG penetrates the root in cut-and-flow experiments, its axial flux has the same form: *J*_peg_=*JC*_peg_ (mol s^–1^) where *C*_peg_ is the PEG concentration in the xylem vessels. Details about the discretization of the equations and their resolution on the matrix form of the RSA can be found in Supplementary Protocol S1.

The overall coupled model of solute and water transport within an RSA is summarized in Fig. 1. Figure 1C sketches a case where the hydrostatic pressure of the bathing solution is greater than the atmospheric pressure at the basal part of the root, and the outer concentration of solutes is lower than that of the sap. Added to the radial water flux, the two solute transport components (*J*_s_* and *P*_s_) are also represented.

### Viscosity and water potential of a PEG solution

The water potential of a PEG solution, Ψ_peg_, does not follow Van’t Hoff’s law but can be described, at a given temperature, by an empirical polynomial law (see equation 1 in Michel, 1983 and Supplementary Fig. S1A). The osmotic pressure of the PEG solution can be expressed as π_peg_= –Ψ_peg_. For a PEG concentration of 150 g l^–1^, Ψ_peg_= –0.302 MPa.

To consider the viscosity (μ) of a PEG solution (Supplementary Fig. S1B), we used the following law:


$$\begin{array}{l} µ\left(w_{peg}\right)=-17.4+18.4\;exp\left(\frac{w_{peg}}{0.279}\right) \end{array}$$

which derives from a fit done on data from Gonzalez-Tello *et al.* (1994) for w_peg_ between 100 g l^–1^ and 200 g l^–1^, with a data point (1 mPa s) added at w_peg_=0 to integrate the viscosity of pure water. Note that a PEG solution at 150 g l^–1^ exhibits a viscosity of 14 mPa s while the viscosity of water is 1 mPa s.

### Parameter determination

The principle is to adjust the axial conductance (*K*) profile, the radial hydraulic conductivity (*k*), the active solute uptake (*J*_s_*) and the solute permeability (*P*_s_) to get the best fit on both *J*_v_(*P*) and cut-and-flow experiments. The fit is obtained by minimizing the objective function *F* set as the sum of the squared errors, *F*=Σ(*J*_v_–*J*)^2^, with *J*_v_ being the experimental data and *J* the simulated data.

The axial conductance is known to vary with the distance to the root tip (Frensch and Steudle, 1989; Doussan *et al.*, 1998b; Zarebanadkouki *et al.*, 2016; Meunier *et al.*, 2018; Heymans *et al.*, 2021; Boursiac *et al.*, 2022a). The *K* profile was therefore represented as a linear piecewise function of the distance to the root tip. The number of points and their abscissa were the number of cuts and their distance to the tip, respectively. Thus, the function was different between plants, with up to nine points. Since *k*, *J*_s_*, and *P*_s_ are uniform, the maximum number of parameters was 12.

The parameter first guesses used to start the adjustment were as follows. (i) An axial conductance profile derived from the tap root profile of Heymans *et al.* (2021). This profile corresponds to a step function with *K*_min_=10^–12^ and *K*_max_=10^–10^ m^4^ MPa^–1^ s^–1^. The step was positioned at around 0.1 m depending on the abscissa of the cut-and-flow data. (ii) A radial conductivity equal to 10^–7^ m MPa^–1^ s^–1^. This arbitrary value is consistent with literature reports of from 0.5–1.5 × 10^–7^ MPa^–1^ s^–1^ (Heymans *et al.*, 2021) to 2.5 × 10^–7^ m MPa^–1^ s^–1^ (Frensch and Steudle, 1989). (iii) *J*_s_*=10^–7^ mol m^–2^ s^–1^ and *P*_s_=10^–9^ m s^–1^. Such orders of magnitude can be found in the literature for maize or *Phaseolus* roots (Fiscus, 1986; Steudle and Brinckmann, 1989; Birner and Steudle, 1993).

We used the optimize.minimize function of the SciPy Python library (Virtanen *et al.*, 2020) to perform these minimizations. Local optimization routines were used because global optimizations, such as dual annealing, were often unable to get a correct fit. Due to the heterogeneity of the plants and condition panel, an identical numerical workflow was sometimes not possible. Thus, we used different optimization routines, running the optimization process several times. The main workflow was: (i) to start from the first guesses with the adjustment of *K* and *k* on the cut-and-flow data without any solute transport using the workflow of Boursiac *et al.* (2022a); (ii) to keep the *K* obtained in (i), adjusting *k*, *J*_s_*, and *P*_s_ on the *J*_v_(*P*) data—here, the solver of optimize.minimize that was mainly used was sequential least squares programming (SLSQP); and (iii) to finalize with the adjustment of all the parameters on *J*_v_(*P*) and cut-and-flow data—here, the solver that was mainly used was constrained optimization by linear approximation (COBYLA).

## Results

### J_v_(*P*) and cut-and-flow experiments in roots under control and water deficit conditions

Focusing on functional responses of maize primary roots to WD, the pressure chamber technique was used to measure the *J*_v_(*P*) and obtain cut-and-flow data in de-topped roots. WD was induced by addition of PEG 8000 (150 g l^–1^), lowering the water potential of the bathing solution to –0.336 MPa. Three different sets of growth and experimental conditions were used. Control (CTR) plants were grown in a standard hydroponic solution and de-topped roots were studied in the same solution. PEG plants were exposed to WD for 5 d with water transport measurements done in the same PEG bathing solution. PEG-CTR roots were derived from plants grown in a PEG solution and transferred into a control solution for 1 h prior to pressure chamber measurements in a control solution. Besides methodological considerations, the latter treatment allows exploration of the reversibility of WD effects on root water transport parameters.

**Fig. 2.**
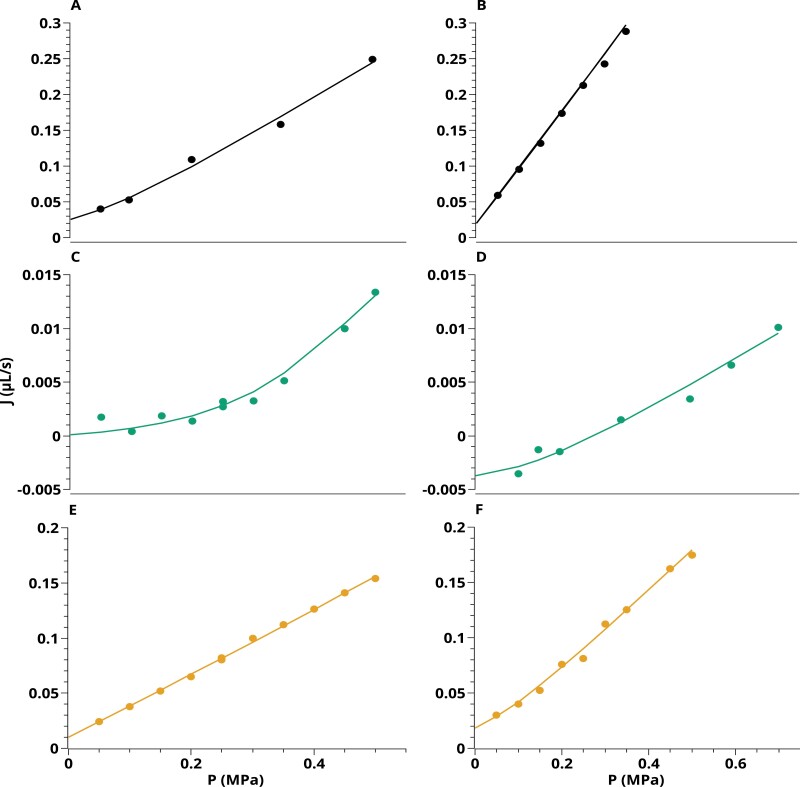
Representative examples of *J*_v_(*P*) curves in individual primary roots. (A, B) CTR roots. (C, D) PEG roots. (E, F) PEG-CTR roots. Circles represent experimental data, whereas solid curves indicate the best fit obtained with the model.

**Fig. 3.**
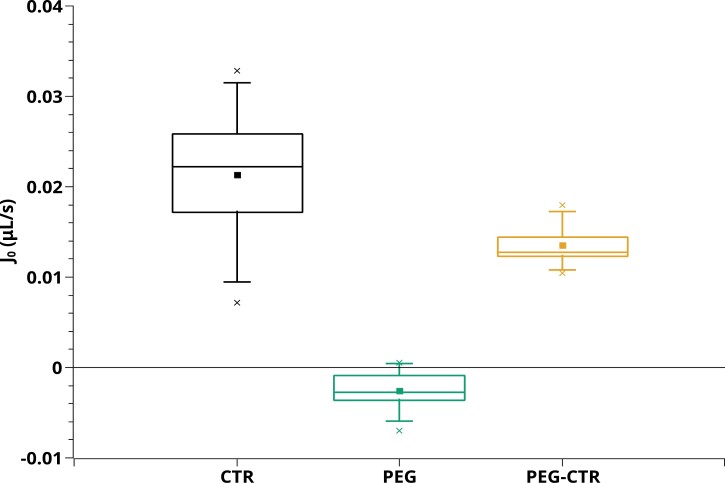
Flow of xylem sap exuded from excised roots at atmospheric pressure. CTR (black), *n*=8; PEG (green), *n*=8; PEG-CTR (orange), *n*=5. Each box indicates the 25th and 75th percentiles, while the line inside indicates the median value, and the T bars mark the fifth and 95th percentiles.

Figure 2 shows six examples of *J*_v_(*P*) relationships measured in the three series of roots. PEG roots (Fig. 2C, D) showed non-linear curves, a property observed in six out of eight plants. In contrast, *J*_v_(*P*) curves of the two other root types (CTR and PEG-CTR) were more linear. Yet, two of these roots (Fig. 2A, F) showed a weak non-linearity below 0.1 MPa. Another key property emphasized by Fig. 2D is the possible occurrence of negative xylem flow rates for positive hydrostatic pressure differences, up to 0.2 MPa. This is also shown in Fig. 3, which displays xylem flow rates at atmospheric pressure [*J*_0_=*J*_v_(0)], without any external hydrostatic driving force. These flow rates are very stable over time and could be measured in some roots for up to 1 h. For the eight PEG roots, the average *J*_0_ was –2.5 ± 0.9 10^–3^ μl s^–1^. In contrast, roots bathing in a control hydroponic solution yielded positive *J*_0_ values (CTR: *J*_0_=2.1 ± 0.8 × 10^–2^ μl s^–1^; PEG-CTR: *J*_0_ = 1.4 ± 0.6 × 10^–2^ μl s^–1^) (Fig. 3).

**Fig. 4.**
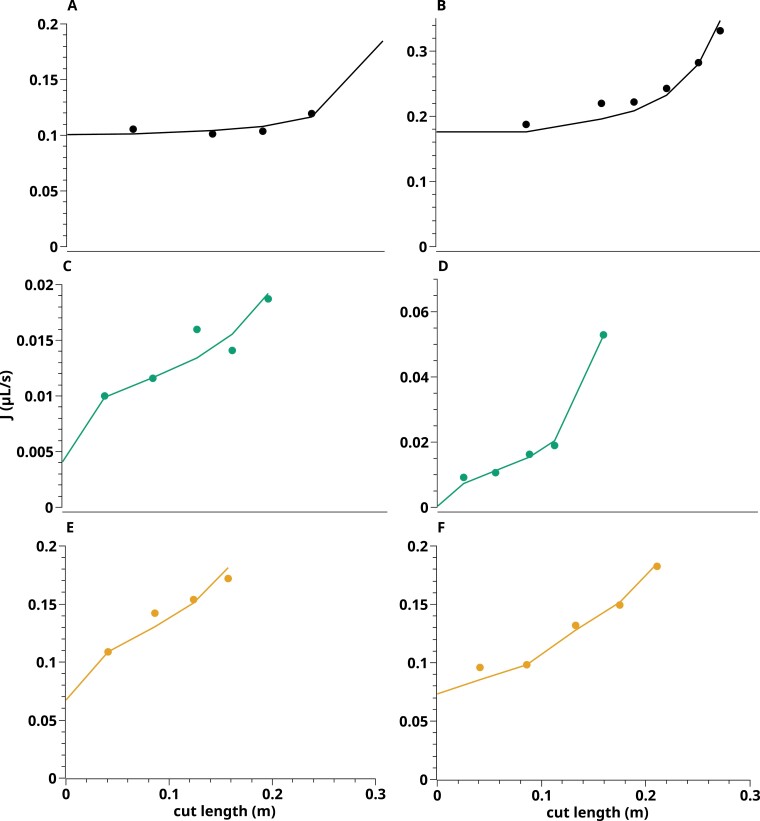
Representative examples of cut-and-flow data in individual primary roots. The data are derived from the same roots as in Fig. 2. (A, B) CTR roots. (C, D) PEG roots. (E, F) PEG-CTR roots. Circles represent experimental data, whereas solid lines indicate the best fit obtained with the model.

Figure 4 is the counterpart of Fig. 2. It refers to the same individual roots, showing corresponding cut-and-flow experiments performed after *J*_v_(*P*) measurements (Fig. 2). In brief, Fig. 4 shows increases in pressure-induced flow rate upon progressive cuts of the root system from root tips to base. The curves determined in CTR roots are characterized by a low initial slope that increases with length of the cut root (Fig. 4A, B). The four other curves, derived from PEG and PEG-CTR roots, show a less convex shape, with higher slopes from the first cut. Thus, the cut-and-flow curves ­capture the impact on root functional properties of plant growth under WD.

The same conclusions can be drawn from *J*_v_(*P*) and cut-and-flow data obtained in a total of 15 additional CTR, PEG, or PEG-CTR roots (Supplementary Figs S2–S4).

### Integration of solute and water flows throughout RSA

**Fig. 5.**
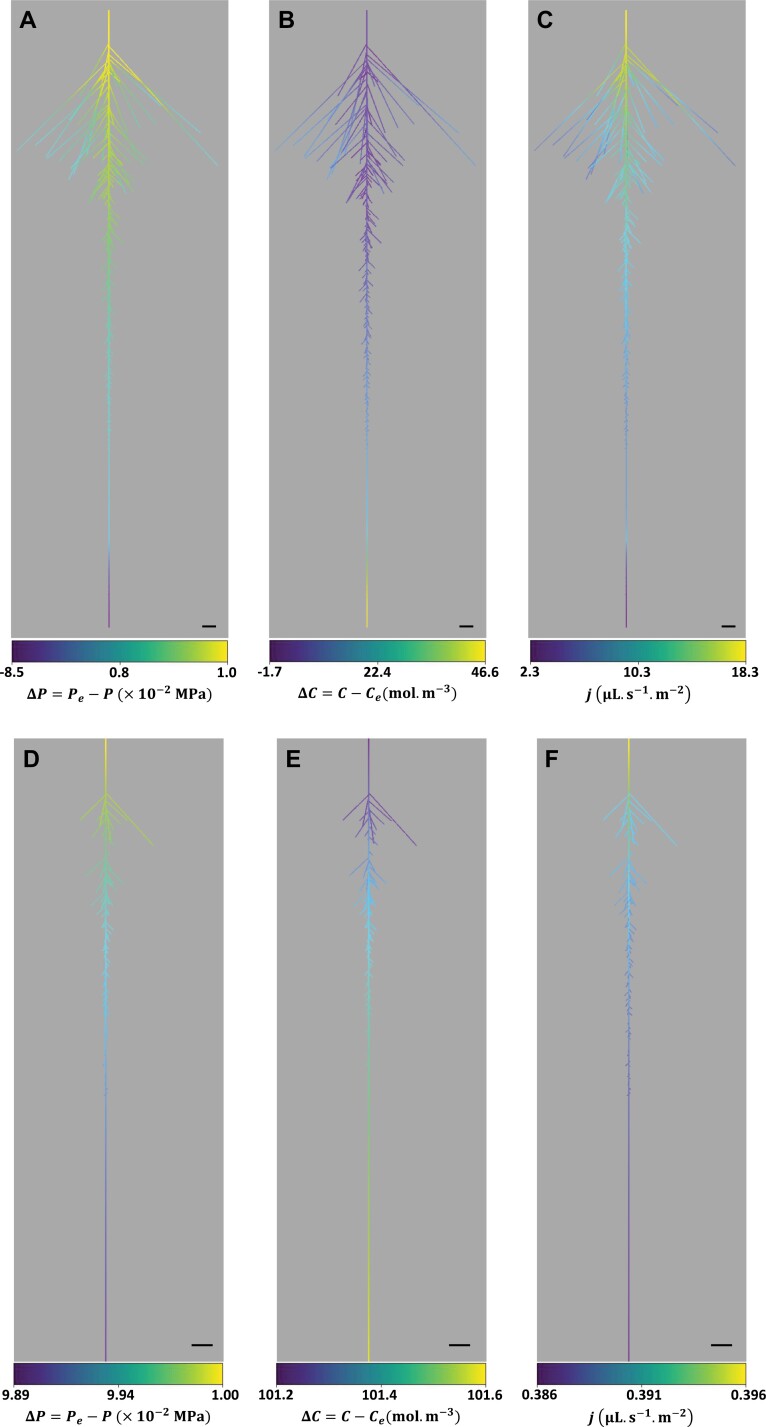
Heat maps of driving components and radial water flux throughout the RSA of a CTR root (A–C), and a PEG root (D–F) bathing in a CTR or PEG solution, respectively. In both cases, a hydrostatic overpressure of 0.1 MPa was applied to the external medium. Δ*P*=*P*_e_–*P* is the difference between the hydrostatic pressure of the external medium, *P*_e_, and the xylem vessel pressure *P*, in MPa (A and D). Δ*C*=*C–C*_e_ is the difference between solute concentration in the xylem vessels, *C*, and the external concentration, *C*_e_, in mol m^–3^ (B and E). *j* is the local radial flow of water in µl s^–1^ m^–2^ (D and F). Scale bar=10 mm.

Based on the experimental data documented above, we developed an RSA model of water and solute transport allowing fitting of the *J*_v_(*P*) and cut-and-flow data of roots exposed to control or WD conditions (Figs 2, 4; Supplementary Figs S2–S4). Indeed, a pure water transport model could not explain exudation at atmospheric pressure or negative fluxes observed in some PEG roots. Basically, an osmotic term was added to the radial water flux (Equations 1 and 2) and solute transport was coupled to hydraulic flow. Using the model within a realistic RSA allowed mapping of the heterogeneity of solute and water flows throughout the root. Figure 5 shows representative CTR (Fig. 5A–C) and PEG (Fig. 5D–F) roots bathing in a CTR or PEG solution, respectively, under the same hydrostatic overpressure of 0.1 MPa. Note that, as indicated below, the hydraulic and solute transport parameters were determined by fitting *J*_v_(*P*) and cut-and-flow data (Figs 2A, C, 4A, C). For each root, the figure depicts the heat map of the hydrostatic and osmotic driving components (see Equation 2), Δ*P*=*P*_e_–*P* and Δ*C*=*C*–*C*_e_, and the resulting local radial water fluxes. The CTR root shows an increasing Δ*P* from the tips to the base, with a negative difference at the primary tip that would drive a radial efflux of water (Fig. 5A). Yet, influxes occur throughout the RSA (Fig. 5C), indicating that the dominating force driving water influx in the primary tip is Δ*C* (Fig. 5B). In addition, a progressive drop in xylem solute concentration is observed along the RSA, due to dilution effects by the sap flow. Thus, the progressive increase in radial flows along the RSA can be explained by dominating effects of variations in Δ*P* over variations in Δ*C* (Fig. 5C). Although the PEG root is bathing in a medium with a low osmotic potential (–0.336 MPa), it presents the same trends as the CTR root, with water influxes throughout the RSA, a progressive increase of Δ*P* from the tips to the base, and, inversely, a progressive drop of Δ*C* (Fig. 5D–F). Whereas radial water influx varies in parallel to Δ*P* variation, as in the CTR root, the absolute value of Δ*P* remains in the range of 0.1 MPa which is insufficient to counter-balance the external water potential of –0.336 MPa. Therefore, both Δ*P* and Δ*C* serve as joint driving forces. Finally, we note that, although the PEG root harbors gradient properties throughout its architecture, these variations are of very low amplitude (<1%), much lower than in the CTR root [–0.085<Δ*P*<0.100 (MPa); –1.7<Δ*C*<46.6 (mol m^–3^)]. Therefore, the PEG root has much more homogeneous properties inside the xylem vessels than the CTR root.

**Fig. 6.**
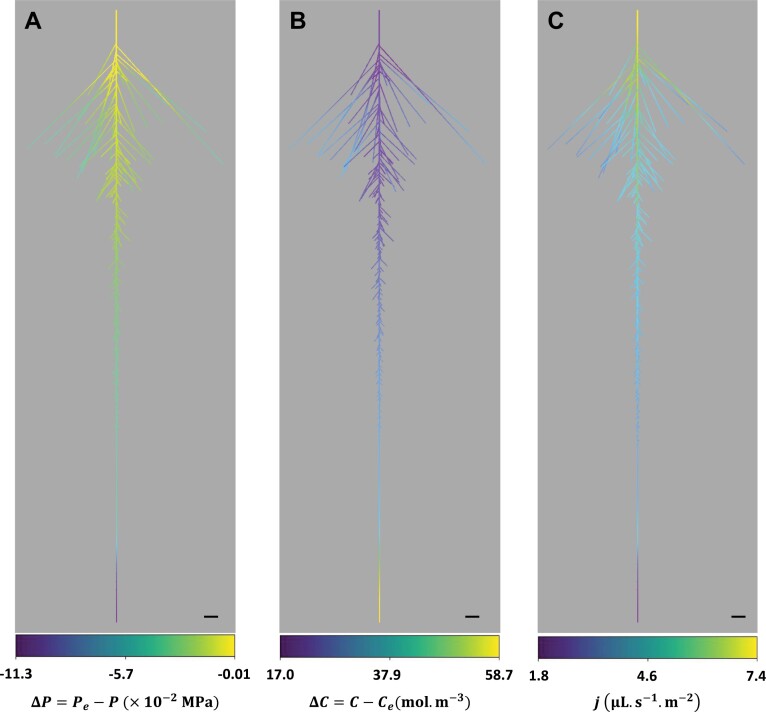
Heat maps of driving components and radial flux throughout the RSA of a CTR root in a CTR solution and in conditions of spontaneous exudation (the bathing solution was at equilibrium with atmospheric pressure). The notations and conventions are the same as in Fig. 5. Scale bar=10 mm.

Figure 6 shows the heat maps of Δ*P*, Δ*C*, and local radial fluxes in a representative CTR root, but under a spontaneous exudation condition. A water influx can be observed throughout the RSA (Fig. 6C). Yet, a negative Δ*P* can be observed over the whole architecture, with an increasing value to the base (Fig. 6A). Such Δ*P*, which counteracts radial water inflow, is due to the induction of a sap flow in the xylem. As discussed above, the Δ*C* decrease to the tips results from dilution effects due to water flow (Fig. 6B). Therefore, the major driving force for radial influx is Δ*C*, and its variation along the RSA is concomitantly controlled by Δ*P* and Δ*C*.

These few examples illustrate the capacity of the present modeling approach to resolve how integration throughout an RSA of discretized elementary modules leads to heterogeneity in driving forces and elementary flows, depending on both root type and water transport mode.

### Axial conductance profiles of roots under standard and WD conditions

The present approach was further used to obtain a comprehensive view, using model inversion, of the water and solute transport properties of real roots, obtained from CTR or PEG plants, and measured in CTR or PEG solutions. Here again, parameter adjustments were done concomitantly on the *J*_v_(*P*) and cut-and-flow data, taking into account penetration of PEG in xylem vessels during cut-and-flow experiments. One set of parameters was therefore obtained for each of 21 roots investigated. Best fits of these experiments are shown in Figs 2 and 4 and Supplementary Figs S2–S4. We note that, for all roots investigated, the goodness of fit (*R*^2^) for the relationship between the measures and values simulated from the fitted model was >0.91 (Supplementary Fig. S5).

**Fig. 7.**
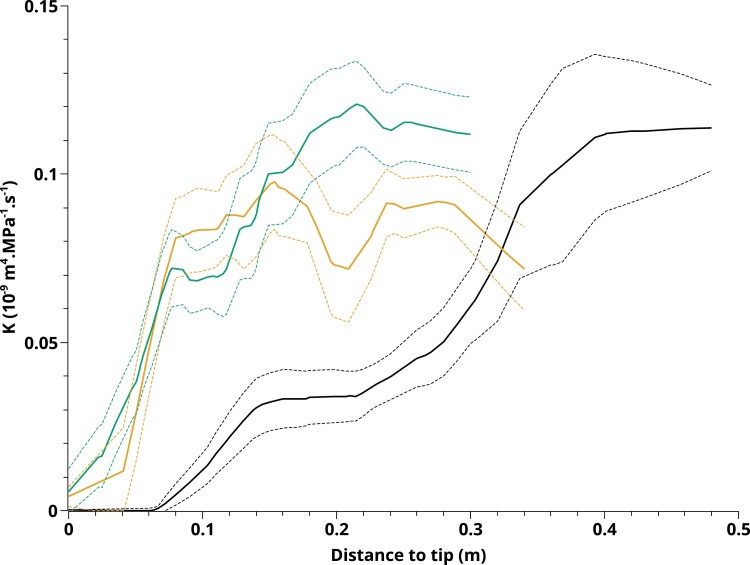
Variations of axial conductance (*K)* as a function of distance to root tip. The solid lines represent lowess fits done on *K* profiles of CTR roots (black; *n*=8), PEG roots (green; *n*=8), and PEG-CTR roots (orange; *n*=5). The dashed lines delineate the corresponding 95% confidence intervals.

The axial conductance (*K*) profiles obtained by locally weighted scatterplot smoothing (lowess) of CTR (*n*=8), PEG (*n*=8), and PEG-CTR (*n*=5) datasets are shown in Fig. 7. The *K* profiles of roots grown in the presence of PEG, whatever the *J*_v_(*P*) measuring solution (PEG or CTR), are very close to each other. Since measurements in PEG-CTR roots were performed 1 h after their transfer into a CTR solution, we assume that their xylem anatomy (and therefore their *K* profile) was similar to that of PEG roots. Thus, these results indicate that the bathing solution used during the cut-and-flow experiment, and in particular the presence or absence of PEG, does not interfere with the determination of *K*. As a consequence, our approach reveals, with respect to CTR roots, a dramatic increase of *K* in root tips grown under WD.

### Determination of radial hydraulic conductivity and solute transport parameters

**Fig. 8.**
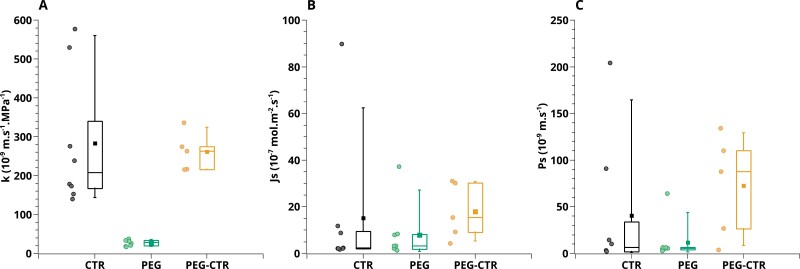
Values of radial hydraulic conductivity (A), active solute uptake rate (B), and solute permeability (C) determined by inverse modeling in the indicated root types. Each box indicates the 25th and 75th percentiles, while the line inside indicates the median value, and the T bars mark the fifth and 95th percentiles. Corresponding individual values are shown with the same color on the left of each box plot.

Figure 8 shows the distribution of the three radial transport parameters giving the best fits. With respect to CTR roots, PEG roots showed a 10-fold decrease in mean radial hydraulic conductivity (*k*). This result is in accordance with the decrease in hydraulic conductivity commonly observed in roots under WD (Steudle, 2000b), such as in Arabidopsis grown in a 150 g l^–1^ PEG solution (Rosales *et al.*, 2019). PEG-CTR roots showed *k* values similar to those of CTR roots, pointing to a quick reversal of the WD-induced inhibition of *k*. The solute transport parameters (*J*_s_*, *P*_s_) were more difficult to interpret with respect to known effects of WD. This difficulty was reflected by the fact that the CTR and PEG-CTR data were very scattered.

### Sensitivity analysis of the model

To possibly understand the scattering of *J*_s_* and *P*_*s*_ inferred values, we investigated the sensitivity of adjusted *k* to the two solute transport parameters. In the following, we note that *k*_0_, *J*_s0_*, and *P*_s0_ are the parameter values giving the best fit. The test was run for the CTR and PEG roots corresponding to Fig. 2A and C, respectively. It consisted of adjusting *k* on *J*_v_(*P*) for various values of *J*_s_* and *P*_s_ and looking at the range of *J*_s_* and *P*_s_ variations for a 10% change of *k* around *k*_0_. For the CTR root, a ±10 % variation of the adjusted *k* was obtained for *J*_s_ varying from 5 × 10^–4^ to four times *J*_s0_ and for *P*_s_ varying from 5 × 10^–4^ to 131 times *P*_s0_. The objective function (*F*) was almost multiplied by 10, varying from its minimum 2.6 × 10^–4^ to 2.2 × 10^–3^. In the PEG root, a ±10% change of the adjusted *k* was obtained for *J*_s_ varying from 4 × 10^–4^ to 2.05 times *J*_s0_ and for *P*_s_ varying from 1 × 10^–3^ to 4.0 times *P*_s0_, with *F* varying by two orders of magnitude from its minimums. Thus, in either type of roots, the determination of radial hydraulic conductivity *k* shows low sensitivity to the active uptake rate and the passive permeability of solutes.

In the present model, the reflection coefficient (σ) was initially set to 0.85, a value previously reported for nutrients in maize root systems (Miller, 1985a). In order to evaluate the impact of σ on determination of other parameters, we performed two sets of adjustment, with two contrasting values, σ=1 and σ=0.5, respectively. With respect to axial conductance profiles determined with σ=0.85, the maximum discrepancy was obtained for CTR roots and σ=1, with an average change of *K* of 1.2%. In all the other cases, *K* variations were lower than 1%. Determination of *k* was also largely insensitive to σ, with a maximum change of mean *k* below 3% (Supplementary Fig. S6A, D). For the two other parameters, *P*_s_ and *J*_s_*, the results were more contrasted (Supplementary Fig. S6B, C, E, F). In CTR and PEG roots, mean *J*_s_* and *P*_s_ could increase by up to 25% (σ=0.5). In PEG-CTR roots, mean *J*_s_* and *P*_s_ were increased by up to 40% and 6%, respectively, for σ=0.5.

These analyses assess the accuracy with which effects of WD on root hydraulic parameters can be determined using the present inverse modeling approach.

## Discussion

In the present work, we developed a model of root hydraulic architecture aimed at describing the behavior of maize primary roots under control or WD conditions. Besides radial and axial water transport, that were represented using a fairly classical hydraulic tree model, solute transport was also considered throughout RSA, using both an active uptake rate and a tissue permeability coefficient. As in our earlier work (Boursiac *et al.*, 2022a), our main aim was to develop an inverse modeling approach to assess the radial conductivity and the axial conductance profile of a branched root system. Yet, this procedure was improved as it relied not only on sap flow data obtained in cut-and-flow experiments but also on extensive *J*_v_(*P*) data obtained in intact roots. Most importantly, this procedure was validated in plants grown under standard conditions or PEG-induced WD. We showed that the solute transport must be considered to explain sap exudation at atmospheric pressure, when the driving force is essentially osmotic. Solute transport also allows fitting of the specific features of the *J*_v_(*P*) data of some plants under WD with a non-linear relationship and negative sap fluxes. The experiments performed in different conditions demonstrated the robustness of the method to determine concomitantly the radial and axial water transport parameters on real RSA. Most importantly, they allowed us to obtain a comprehensive view of combined effects of WD on radial and axial hydraulic conductances.

### The *J*_v_(*P*) curve shape

The *J*_v_(*P*) data reported in the present work showed two key properties: negative sap flow rates specific to PEG roots, and a non-linearity that was particularly accentuated in PEG roots. A non-linearity of sap flow according to applied pressure has already been described in maize (Miller, 1985b; Gorska *et al.*, 2008) or other plant species (Fiscus, 1986; Markhart and Smit, 1990; Rüdinger *et al.*, 1994; Jackson *et al.*, 1996). For instance, Miller (1985b) reported *J*_v_(*P*) curves that were linear for positive relative pressures but showed a non-linear shape at negative relative pressures. Gorska *et al.* (2008) observed that the *J*_v_(*P*) relationship can be considered as linear only for pressure above 0.1 MPa. These reports are in accordance with our measurements in roots bathing in a CTR solution, whatever the plant growth conditions (CTR or PEG). For roots grown and maintained under WD, we found the non-linearity to be further accentuated. This can be explained by the dramatically reduced radial conductivity of these roots (Fig. 8A). Since the non-linearity of *J*_v_(*P*) results from the changing balance between hydrostatic and osmotic driving forces, the reduced radial conductivity shifts to higher positive pressures the range at which the hydrostatic forces become preponderant and *J*_v_(*P*) linear.

Yet, the non-linearity of *J*_v_(*P*) cannot be modeled without considering solute transport. An absence of solute fluxes would imply that the osmotic pressure term in Equation 2 is uniform over the RSA, leading to a linear relationship. In fact, when *J*_v_(*P*) is monitored at high flow rates, this function can reasonably be adjusted with a linear fit. The slope can then be interpreted as the root hydraulic conductance, which yields the root hydraulic conductivity. In addition, the intercept with the pressure axis, *P*_0_, is usually assumed to be the pressure needed to counter-balance the osmotic pressure of the bathing solution (Passioura, 1988). To explore this further, we tried to determine *K* and *k* using such a purely hydraulic model, exclusively based on Equations 2 and 3. In Equation 2, the osmotic pressure term was set constant and equal to *P*_0_, and therefore Equation 2 was expressed as *j*=*k*(*P*–*P*_0_)*S*, with *P* being the relative pressure. The fits were done on the linear part of *J*_v_(*P*) and on the cut-and-flow experiment. In CTR roots, *k* was found to be reduced by 9% with respect to the value obtained with the water solute transport model. The errors on the *K* axial conductance profile were higher, up to 37%. In PEG roots, the differences between the two model settings were higher, the averaged *k* being reduced by 21% and *K* by up to 84% when the osmotic pressure term was set to *P*_0_. Thus, a simplified osmotic model which does not take solute transport into account results in significant variations in hydraulic parameter estimations. Supplementary Fig. S7 shows best fits of the experiments with a purely hydraulic model, and a constant osmotic pressure *P*_0_. While the quality of fit with CTR roots is good (*R*^2^= 0.95), this type of model is unable to fit the PEG data at low pressure when the osmotic term is preponderant (*R*^2^<0.83).

### Development of a water/solute model at RSA level

Models that couple water and solute transport have been in existence for decades, but they usually represent the whole root as a simplified osmometer with two or three compartments (Fiscus, 1975, 1977, 1986; Miller, 1985b; Steudle, 1994, 2000a). In these models, the root interior (i.e. the inner volume of the xylem vessels) is supposed to have a homogeneous solute concentration and hydrostatic pressure. Consequently, the water and solute fluxes are uniform all over the root surface. This is, however, not the case in real root systems, where sap flow through axial resistances results in a hydrostatic pressure gradient along xylem vessels. This pressure gradient leads to a non-uniform pressure difference between the outer solution and the xylem vessels, that increases from the root tips (laterals and primary) to the basal ends (Figs 5A, D, 6A). The radial flow of water shows the same trend along the RSA (Figs 5C, F, 6C). Consequently, the sap flow rate increases towards the root base, leading to a progressive decrease of the solute concentration (Figs 5B, E, 6B). In osmometer models, this dilution along the RSA is typically neglected. Consequently, these models overestimate the solute concentration inside the xylem compartment, leading to an overestimation of the flow rate. Indeed, Δ*C*=(*C*_e_–*C*) decreases with increasing (overestimated) *C* and, as a consequence, *J*=*k*(Δ*P*–*RT*Δ*C*) increases in these conditions.

To illustrate this point, we calculated an analytical solution of the present model, based on Equations 2 and 4, for a root represented according to Fiscus (1975, 1977) as a simple semi-permeable barrier separating two compartments (see Supplementary Protocol S2). The comparisons of the adjusted transport parameters between the RSA model and the two-compartment model show marked differences for CTR and PEG-CTR roots (Supplementary Fig. S8). In particular, the radial conductivity *k* is significantly underestimated in the two-compartment model, by 65% for CTR roots and 45% for PEG-CTR roots. In contrast, the solute parameters, *J*_s_* and *P*_s_, increase dramatically, by 77% and 78%, respectively, for CTR roots, and by 84% and 88%, respectively, for PEG-CTR roots. The water and solute transport parameters were noticeably less affected in PEG roots, with changes between 10% and 18%. This is due to more homogeneous concentration profiles inside these roots because of lower fluxes. For instance, the difference in solute concentration, calculated from the basal end to the tip of the primary root, and divided by its length, was 92.7 ± 29.6, 2.3 ± 0.7, and 12.5 ± 3.0 mol m^–4^, for the CTR, PEG, and PEG-CTR roots, respectively.

### Robustness of the cut-and-flow method to estimate axial conductance in roots under water deficit

As discussed above, the coupling of solute and water transport in the present root model prevented significant errors in hydraulic parameter determination. In addition, the model had to represent the possible inflow of PEG in the root vasculature during cut-and-flow experiments. Effects of PEG on both radial osmotic potential differences and sap viscosity had to be modeled throughout RSA.

To assess these additional modeling constraints, we closely inspected the physiological consistency of data obtained in plants exposed to WD. In brief, both PEG-CTR and PEG plants were submitted to WD during their growth. Yet, the roots of the former plants were exposed to a CTR hydroponic solution for 1 h prior to water transport experiments. In contrast, roots of the latter plants were maintained in a PEG bathing solution during measurements. The axial conductance profiles *K* of the two series of roots were notably similar. In contrast, and in comparison with PEG roots, the radial conductivity of PEG-CTR roots was reversed to CTR root values. We believe this to be coherent, since *K* is linked to the xylem vessels that are fixed structures and probably cannot be altered significantly within 1 h in a CTR solution. In contrast, *k* is related to aquaporin activity that can be reversed within this time lapse.

Whereas our results are in line with studies in numerous species showing WD-induced inhibition of aquaporin activity (Maurel *et al.*, 2015; Rosales *et al.*, 2019), they are at variance with the lack of effects of a PEG-induced WD on maize *L*p_r_, as reported by Hachez *et al.* (2012). Future work will be required to determine to what extent differences in experimental conditions can account for this discrepancy. The precise description of effects of WD on axial conductance also represents a significant advance which will have to be related to effects of drought on root growth and/or xylem differentiation (Ramachandran *et al.*, 2020). Note that the consistency of axial conductance determination would not have been achieved without considering in detail the viscosity of PEG-containing sap. To illustrate this, let us consider Hagen–Poiseuille’s law which predicts that the conductance of a pipe is inversely proportional to the fluid viscosity. Thus, the axial conductance of a pipe filled with a PEG solution at 150 g l^–1^ is 14 times lower than that of the same pipe filled with water (Supplementary Fig. S1B). Indeed, we checked that interpreting the cut-and-flow experiments done in a PEG bathing solution with a sap viscosity of 1 mPa s leads to an underestimation of *K* by about an order of magnitude.

### Reflection coefficient

In agreement with the idea that the maize root is permeable to solutes, we set σ to 0.85 in the present root functional model, a value reported in the literature for complete root systems bathing in a nutrient solution (i.e. the nature of the solutes is unknown and nutrients account for all solutes present in solution) (Miller, 1985a). Yet, we note that in few other works presenting reflection coefficient values for nutrients (non-differentiated solutes) was σ referenced close to unity (Fiscus, 1986; Knipfer and Fricke, 2010; Knipfer *et al.*, 2021). Nevertheless, we further tested the sensitivity of our model inversion method by performing parameter adjustments for σ=1.0 and 0.5, with 0.5 being the lower bound reported for solutes such as sucrose in maize primary roots (Steudle, 2000a). Our results show that hydraulic parameters were essentially insensitive to σ, and that the two solute parameters were not strongly impacted (Supplementary Fig. S6). These considerations further support the robustness of the model inversion method.

### Conclusion

In the present work, we have extended the toolbox developed by Boursiac *et al.* (2022a), combining experiments and modeling to assess the water and solute transport parameters of maize root systems under WD. The experimental data consisted of two successive measurement sets done with a pressure chamber: *J*_v_(*P*) determination, followed by a cut-and-flow experiment. A critical step was to formalize solute transport to account for the specific shape of the *J*_v_(*P*) curve of plants under WD. Furthermore, maize roots grown under WD were investigated under varying measurements conditions, thereby assessing the robustness of our inverse modeling approach. Thus, this novel procedure allows a concomitant determination of water and solute transport parameters and of their alteration upon exposure to WD. In brief, plants grown under WD exhibited, with respect to plants grown under standard conditions, a notable enhancement of axial conductance (*K*) in root tips. Conversely, the radial conductivity *k* was reduced 10-fold upon direct exposure of roots to WD. Further studies will have to address more complex RSAs considering, for instance, specific hydraulic and solute transport properties in lateral roots and a non-uniform radial conductivity along the root axis. To do so, it may be useful to integrate in the present inverse modeling approach other experimental parameters such as solute concentration in the exuded sap. Finally, our approach can be extended to testing the effects of other environmental factors, such as hypoxia, on water and solute transport in roots.

## Supplementary data

The following supplementary data are available at *JXB* online.

Fig. S1. Physicochemical characteristics of PEG 8000 solutions.

Fig. S2. Experimental data and best fits in six individual PEG roots.

Fig. S3. Experimental data and best fits in six individual CTR roots.

Fig. S4. Experimental data and best fits in three individual PEG-CTR roots.

Fig. S5. Plots of best fitted data obtained by model inversion versus experimental data.

Fig. S6. Water and solute transport parameters according to reflection coefficient value.

Fig. S7. Plots of best fitted data obtained by inversion of a purely hydraulic model versus experimental data.

Fig. S8. Comparison of parameter values adjusted using the root system architecture transport model or a two-compartment model.

Protocol S1. Discretization of transport equations and resolution on a matrix form of the root system architecture.

Protocol S2. Analytical solution of the model for a root represented according to Fiscus.

erac471_suppl_Supplementary_Figures_S1-S8_Protocols_S1-S2

## Data Availability

The data supporting the findings of this study are available from the corresponding author, Christophe Maurel, upon request.
