# Supplementary material for: A root functional–structural model allows assessment of the effects of water deficit on water and solute transport parameters
Source: J Exp Bot. 2022 Dec 14;74(5):1594–608. doi: 10.1093/jxb/erac471 (PMC10010609; doi:10.1093/jxb/erac471)
Supplement: erac471_suppl_Supplementary_Figures_S1-S8_Protocols_S1-S2 [file erac471_suppl_supplementary_figures_s1-s8_protocols_s1-s2.pdf]

Supplementary Figures

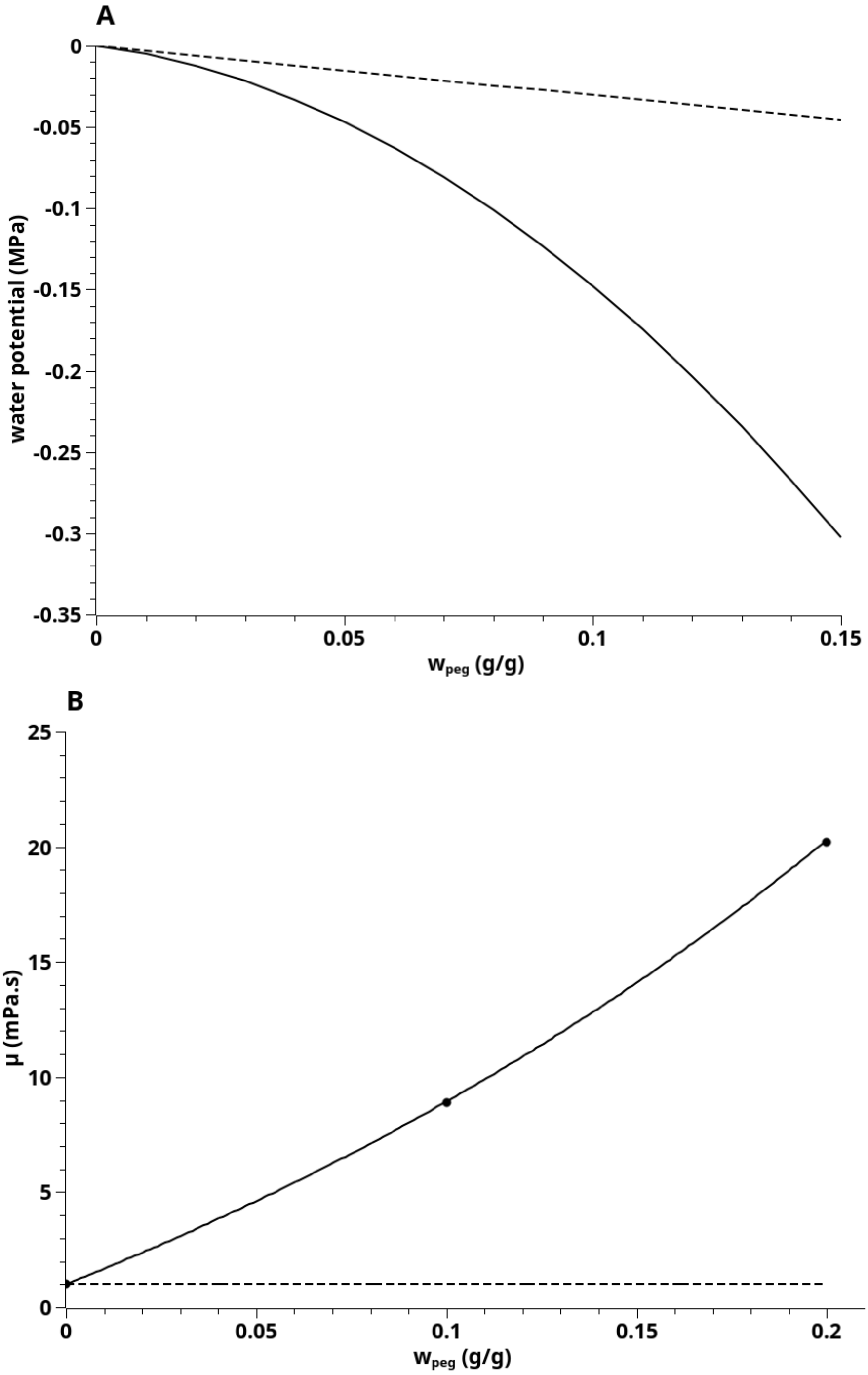

Supplementary Fig. S1: Physicochemical characteristics of PEG 8000 solutions. (A) water potential as a function of PEG concentration (w_{peg}) at 25 °C. The solid line represents the water potential according to equation 1 in (Michel, 1983). The dashed line was derived by calculation using the Van't Hoff's law. (B) viscosity pressure of PEG 8000 solutions as a function of PEG concentration (w_{peg}) at 25 °C. The solid line represents an exponential fit of data (dots) from (Gonzalez-Tello *et al.*, 1994). The dashed line indicates the water viscosity. See Materials and Methods for details.

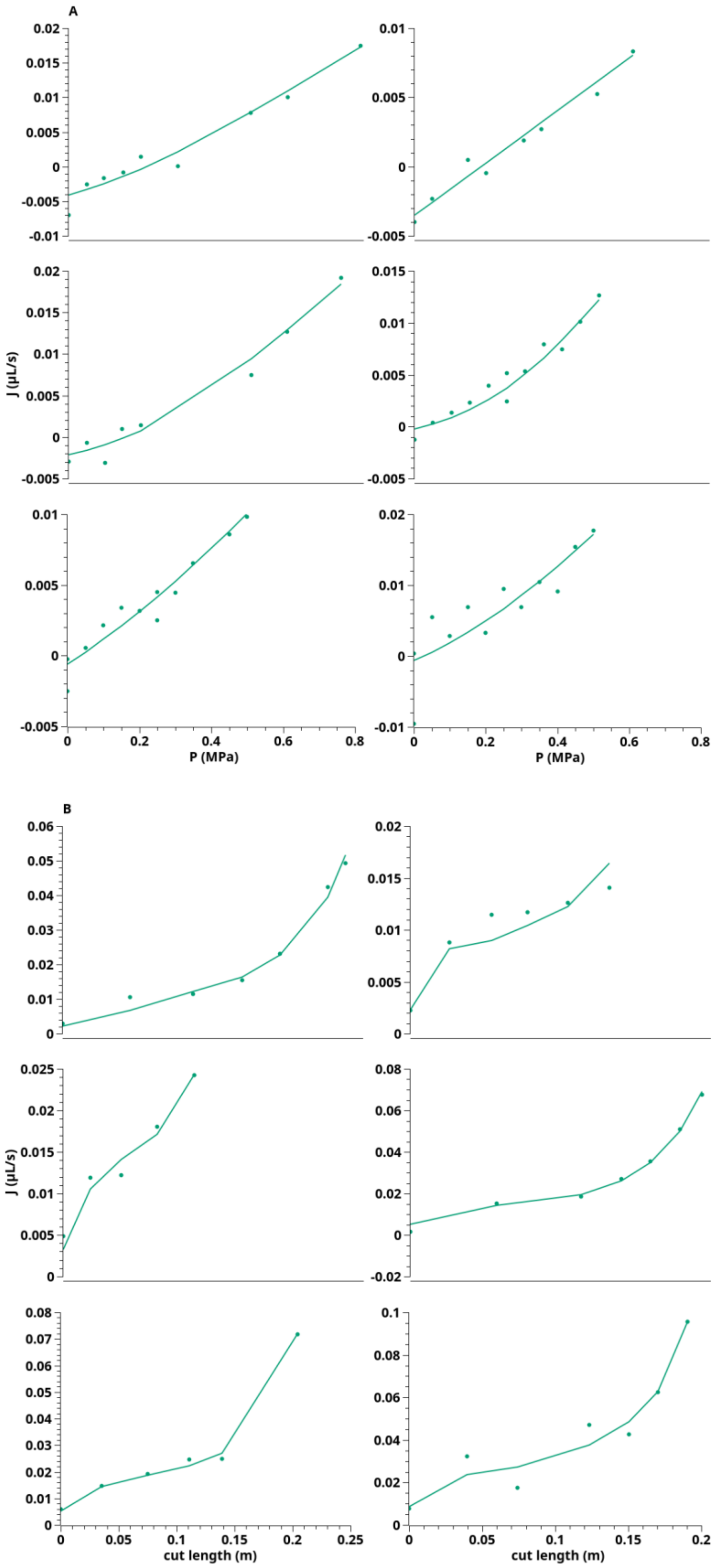

Supplementary Fig. S2: Experimental data and best fits in 6 individual PEG roots. (A) Jv(P). (B) cut and flow. Dots represent experimental data, whereas solid curves indicate the best fit obtained with the model.

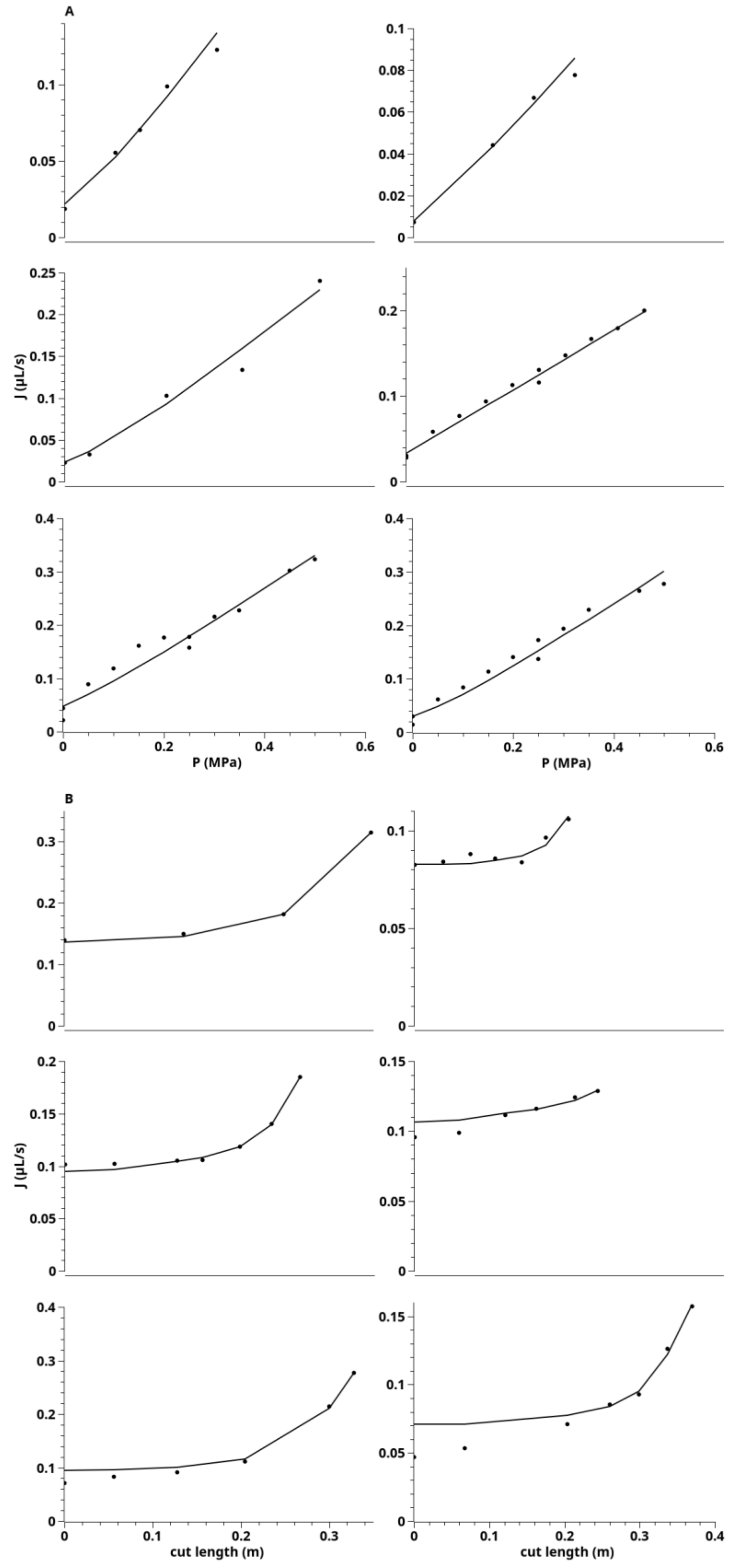

Supplementary Fig. S3: Experimental data and best fits in 6 individual CTR roots. (A) Jv(P). (B) cut and flow. Dots represent experimental data, whereas solid curves indicate the best fit obtained with the model.

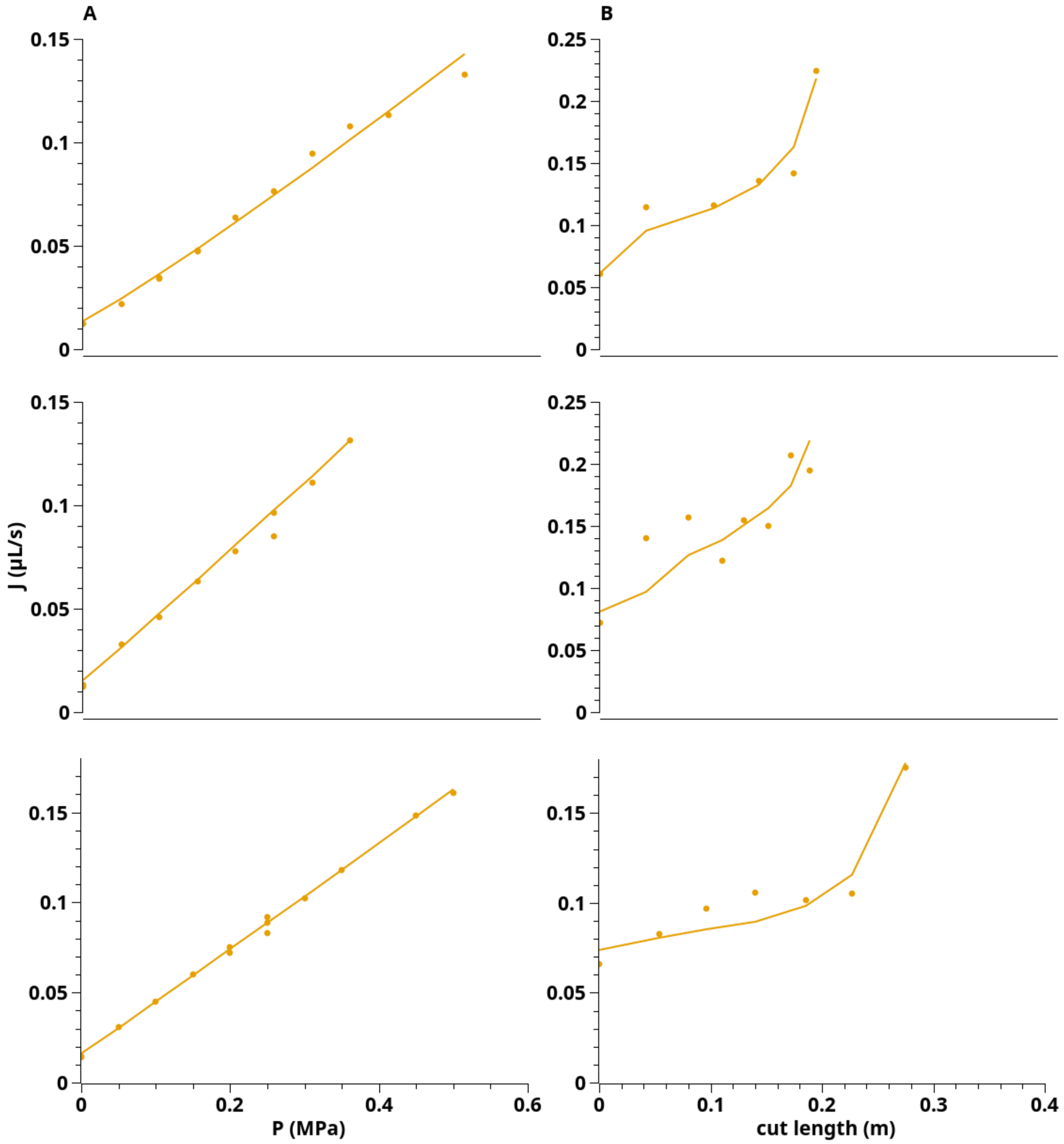

Supplementary Fig. S4: Experimental data and best fits in 3 individual PEG-CTR roots. (A) Jv(P). (B) cut and flow. Dots represent experimental data, whereas solid curves indicate the best fit obtained with the model.

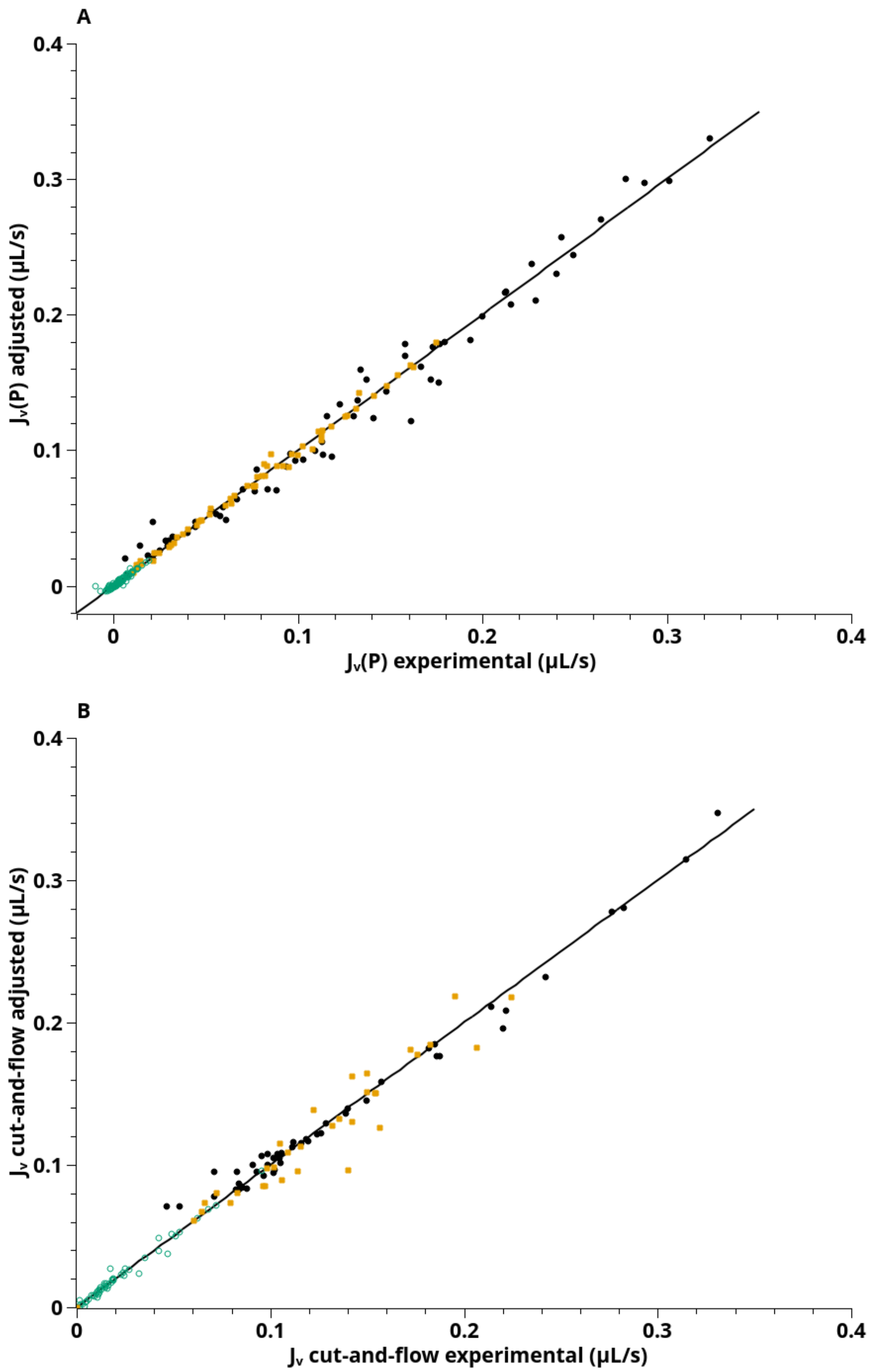

Supplementary Fig. S5: Plots of best fitted data obtained by model inversion versus experimental data. (A) J_v(P). (B) cut-and-flow data. black closed circles: CTR roots; green open circles: PEG roots; orange closed squares: PEG-CTR roots. The black lines correspond to the perpendicular bisector: y=x. R^2 values were as follows: (A) CTR: 9.90 10^{-1}, PEG: 9.80 10^{-1}, PEG-CTR: 9.67 10^{-1}; (B) CTR: 9.79 10^{-1}, PEG: 9.15 10^{-1}, PEG-CTR: 9.94 10^{-1}.

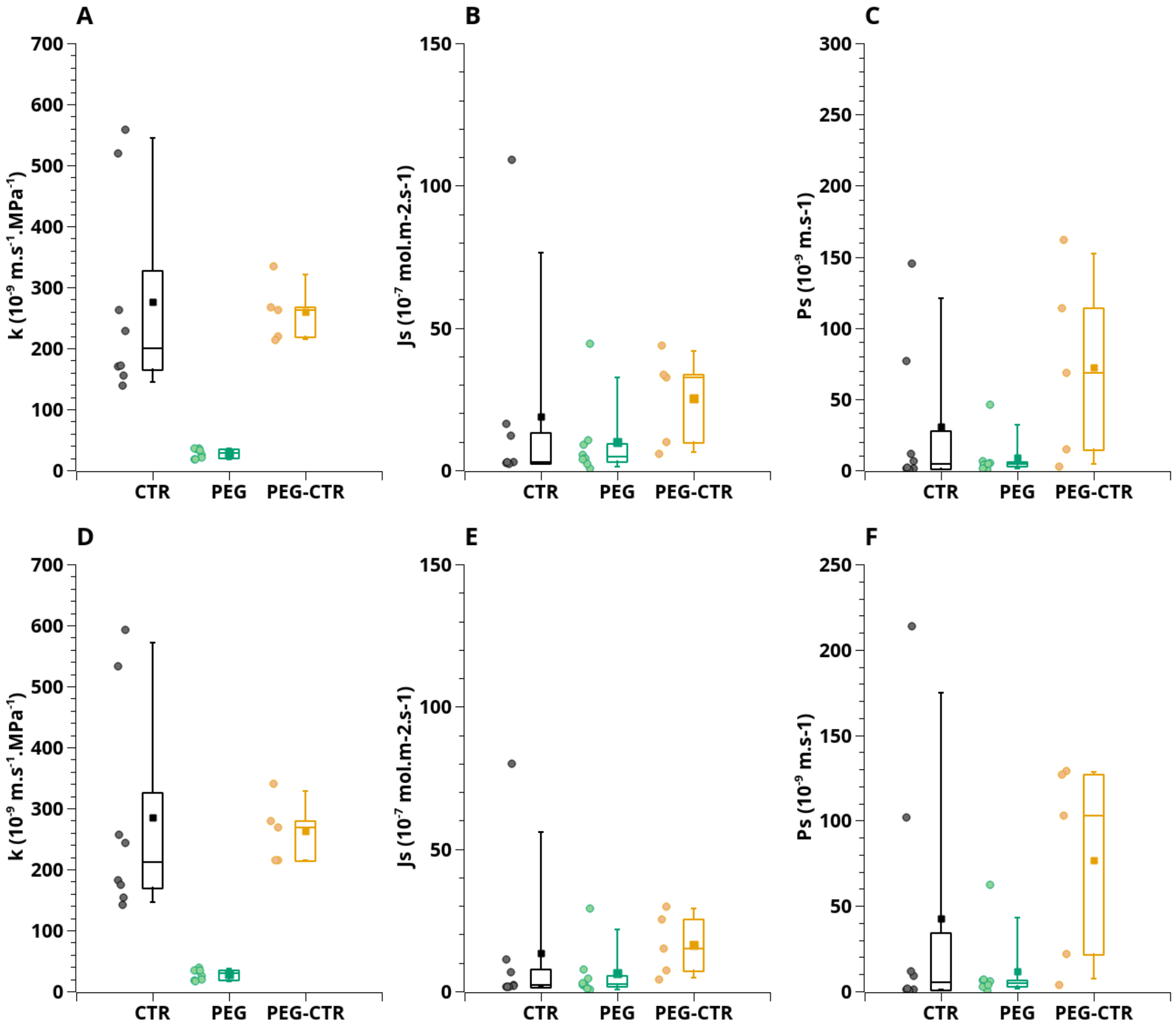

Supplementary Fig. S6: Water and solute transport parameters according to reflection coefficient value. (A-C) σ = 0.5;(D-F) σ = 1. (A, D) radial hydraulic conductivity *k*. (B,E) radial solute flux J_s^*. (C,F) radial solute permeability P_s. Black, green and orange box plots and dots refer to CTR, PEG and PEG-CTR roots respectively.

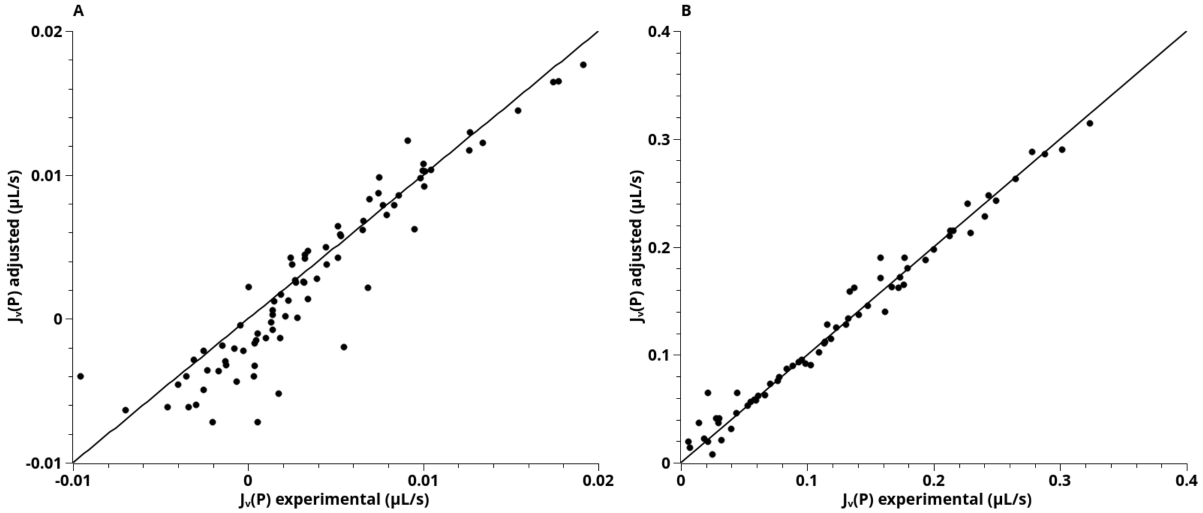

Supplementary Fig. S7: Plots of best fitted data obtained by inversion of a purely hydraulic model versus experimental data. (A) PEG roots. (B) CTR roots. Black closed circles: simulated vs experimental Jv(P) data. The black lines correspond to the perpendicular bisector: y=x. R^2 values were as follows: (A) $R^2 = 8.26\ 10^{-1}$; (B) $R^2 = 9.49\ 10^{-1}$.

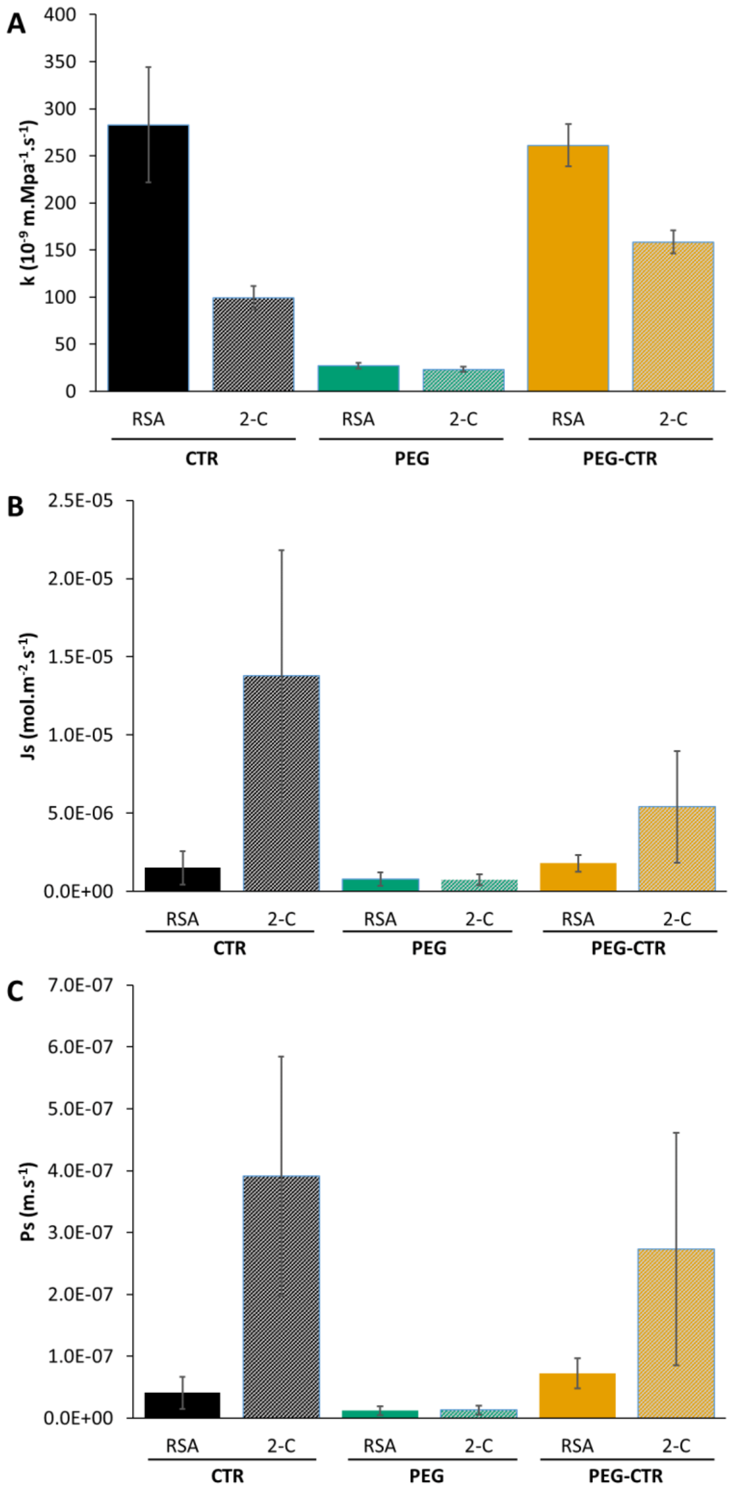

Supplementary Fig. S8: Comparison of parameter values adjusted using the RSA transport model (RSA, plain color) or a two-compartment model (2-C, hatched pattern). (**A**) radial conductivity *k* (**B**) solute uptake rate J_s^* (**C**) solute permeability P_S. Root types were as follows: CTR, black; PEG, green; PEG-CTR: orange. Error bars correspond to SE.

Supplementary Protocols

Supplementary Protocol S1

Discretization of the transport equations:

The root system architecture (RSA) is represented by a Multiscale Tree Graph (MTG) where the nodes are the discretized representation of representative elementary volumes (REV). In the following, REV are numbered from root base to tip.

In each REV, mass conservation is independently applied for water, solutes and PEG. This gives for a REV numbered i:

$$\begin{cases} J_i = \sum_j J_j + k_i \left[P_e - P_i - \pi_{\text{peg}}^{\text{ext}} + \pi_{\text{peg}_i} - \sigma RT(C_e - C_i)\right] S_i \\ J_i \mathrm{X}_i = \sum_j J_j \mathrm{X}_j + [J_s^* - P_s(\mathrm{C}_i - C_e)] S_i \\ J_i \mathrm{X}_{\text{peg}_i} = \sum_j J_j \mathrm{X}_{\text{peg}_j} \end{cases} \quad \text{(A.1)}$$

where P_e, $\pi_{\text{peg}}^{\text{ext}}$ and C_e are the hydrostatic pressure, the osmotic pressure due to the PEG and the solute concentration of the external medium, respectively. The variables with subscript *i* refer to the REV *i*: k_i is the radial hydraulic conductivity, P_i the sap hydrostatic pressure, π_{peg_i} the osmotic pressure corresponding to the local PEG concentration in sap (C_{peg_i}), C_i the solute concentration in sap. S_i is the surface area of the REV. J_i is the outgoing xylem sap flow and J_j is the xylem sap flow coming from node *j*, which stands for one of the children of node *i*. This can be the next node on root axis, or any first node of a lateral root branched on node *i*. k_i is the radial conductivity. σ is the effective reflection coefficient, *R* the gas constant, and *T* the temperature. J_s^* is the solute active uptake rate and P_s is the radial permeability of the root peripheral tissues. J_i and J_j are proportional to the local pressure gradient as follows:

$$J_i = K_i \frac{(P_i - P_{i-1})}{l_i}$$

$$J_j = K_j \frac{(P_j - P_i)}{l_j}$$

with *K* being the axial conductance, *l* the REV length according to the subscript.

X_i is the solute concentration according to the sap flow direction, with $\mathrm{X}_i = \theta_i C_i + (1 - \theta_i) C_{i-1}$, θ_i being a factor that depends on flow direction: $\theta_i = 1$ if $P_i > P_{i-1}$ and $\theta_i = 0$ if $P_i < P_{i-1}$. X_j is the solute concentration flowing between node i and its child j: $\mathrm{X}_j = \theta_j C_j + (1 - \theta_j) C_i$, with θ_j following the same rules as θ_i according to $(P_j - P_i)$. $\mathrm{X}_{\text{peg}_i}$ is the same variable for the PEG concentration.

The third equation of system (A.1) expresses PEG mass conservation at node i.

The (A.1) system can be transformed as follows:

$$
\begin{cases}
G_{\mathrm{w}_i} = J_i - \sum_j J_j - k_i \left[P_e - P_i - \pi_{\mathrm{peg}}^{\mathrm{ext}} + \pi_{\mathrm{peg}_i} - \sigma RT(C_e - C_i)\right] S_i = 0 \\
G_{\mathrm{s}_i} = J_i \mathrm{X}_i - \sum_j J_j \mathrm{X}_j - [J_s^* - P_s(\mathrm{C}_i - C_e)] S_i = 0 \\
G_{\mathrm{peg}_i} = J_i \mathrm{X}_{\mathrm{peg}_i} - \sum_j J_j \mathrm{X}_{\mathrm{peg}_j} = 0
\end{cases}
\qquad \text{(A.2)}
$$

This system can be written in a matrix form as follows:

$$
\mathrm{G}\left(P_i, C_i, C_{\mathrm{peg}_\mathrm{i}}\right) = 0 \qquad \text{(A.3)}
$$

which can be resolved using a Newton-Raphson scheme.

Boundary conditions:

At the base, we consider a Dirichlet boundary condition for the pressure and Neumann boundary condition for the concentration:

$$
P_1 = P_{\mathrm{atm}}
$$
$$
\frac{\partial C}{\partial x} = 0
$$

in other words, root base is at atmospheric pressure and solute and PEG concentrations at the outlet are the same as in the first node.

Supplementary Protocol S2

According to Fiscus (Fiscus, 1975, 1977), we considered the root as a membrane separating two homogeneous compartments and wrote the following analytical solution. At steady-state, the outgoing solute flux equals the entering flux since there are no solute source or sink within the root. Thus, equation (4) yields:

$$
JC = J_s^* S + P_s(C_e - C)S
$$
$$
C = \frac{J_s^* S + P_s C_e S}{J + P_s S}
$$

with *J* the water flow rate, *S* the total surface area of the root, J_S the solute active uptake rate, P_s the radial permeability of the root peripheral tissues, *C* and C_e the solute concentration in the xylem vessels and in the bathing solution, respectively.

Similarly, the entering water flux is equal to the outgoing flux due to mass balance (see equation (2)):

$$
J = k\left(P_e - P - \pi_{\mathrm{peg}}^{\mathrm{ext}} - \sigma RT(C_e - C)\right) S
$$

with *k* the radial conductivity, $\pi_{\mathrm{peg}}^{\mathrm{ext}}$ the osmotic pressure due to the PEG and P_e the hydrostatic pressure in the bathing solution, respectively. *P* is the hydrostatic pressure at the root basal extremity, *σ* the effective reflection coefficient, *R* the gas constant, and *T* the temperature.

Inserting the expression of C we obtain:

$$J^2 + J\left[P_s - k\left(P_e - P - \pi_{\text{peg}}^{\text{ext}} - \sigma RTC_e\right)\right]S$$
$$-kS^2\left[\left(P_e - P - \pi_{\text{peg}}^{\text{ext}} - \sigma RTC_e\right)P_s + \sigma RT\left(J_s^* + P_sC_e\right)\right] = 0 \quad \text{(B.1)}$$

Literature cited

Fiscus EL. 1975. The Interaction between Osmotic- and Pressure-Induced Water Flow in Plant Roots. Plant Physiology **55**, 917–922.

Fiscus EL. 1977. Determination of Hydraulic and Osmotic Properties of Soybean Root Systems. Plant Physiology **59**, 1013-1020.

Gonzalez-Tello P, Camacho F, Blazquez G. 1994. Density and Viscosity of Concentrated Aqueous Solutions of Polyethylene Glycol. Journal of Chemical & Engineering Data **39**, 611-614.
